# The Protective Effects of Burdock Fructooligosaccharide on Preterm Labor Through Its Anti-Inflammatory Action

**DOI:** 10.3390/ijms26062659

**Published:** 2025-03-15

**Authors:** Qunfei Ma, Ruoheng Du, Peihua Long, Kaiyi Sun, Youxia Wang, Ye Yang, Xinyu Shen, Lu Gao

**Affiliations:** 1 Department of Physiology, Naval Medical University, Shanghai 200433, China; maqunfei@smmu.edu.cn (Q.M.); durh@smmu.edu.cn (R.D.); longpeihua@smmu.edu.cn (P.L.); sunkaiyi@smmu.edu.cn (K.S.); wyxesther@smmu.edu.cn (Y.W.); yangye@smmu.edu.cn (Y.Y.); shenxinyu@smmu.edu.cn (X.S.); 2 Shanghai Key Laboratory for Assisted Reproduction and Reproductive Genetics, Shanghai 200120, China

**Keywords:** burdock fructooligosaccharide, preterm labor, myometrium, toll-like receptor 4, inflammation

## Abstract

Most pharmacotherapeutic chemicals/interventions used to manage preterm labor (PTL) often cause neonatal morbidity and maternal adverse reactions. Fructooligosaccharides, extracted from traditional Chinese medicine, can alleviate inflammation, demonstrate antiviral capabilities, and protect against antioxidant stress, implying a potential effective PTL treatment. In this study, we explored the protective effects of the purified burdock fructooligosaccharide (BFO), a Gfn-type fructose polymer, on inflammation-induced PTL. It was found that two doses of 30 mg/kg mouse BFO administration to pregnant mice at a 6 h interval can effectively ameliorate lipopolysaccharide (LPS)-induced PTL. Drug dynamic distribution analysis revealed that BFO was rather highly enriched in myometrial tissues, could inhibit oxytocin-induced uterine smooth muscle contraction, and could bind toll-like receptor 4 (TLR4) on the membrane of uterine smooth muscle cells, downregulating the expression of downstream genes, attenuating the upregulation of inflammatory cytokines in serum and the myometrium, as well as reversing the increased macrophage and neutrophil infiltration into the myometrium induced by LPS. It can also interfere with the levels of estrogen and progesterone, alleviating the occurrence of premature birth. These findings collectively suggest that BFO might serve as a promising therapeutic agent for inflammation-related preterm labor to safeguard the health of both the mother and fetus.

## 1. Introduction

Preterm labor (PTL) refers to childbirth after 28 weeks but less than 37 weeks of gestation, emerging as a substantial contributor to the mortality and morbidity of infants and children younger than 5 years [1,2]. Moreover, premature infants often suffer from complications with potential lifelong impacts on postnatal health and development, including visual and hearing impairments, asthma, attention deficits, and increased anxiety [1].

There are multiple intricate etiologies underlying PTL that make its prevention and treatment quite challenging [3,4]. Among the known etiologies, infections and inflammation account for 20 to 30% of preterm premature rupture of membranes (PPROM) and 25 to 40% of PTLs with intact membranes. Most spontaneous or unexplained PTLs have also been associated closely with infections or inflammation [1,5]. Some bacteria and viruses activate the toll-like receptor (TLR) signaling pathway, leading to the expression of adaptor proteins such as MyD88, TRAF6, and NF-κB. This activation mediates the synthesis of pro-inflammatory mediators, including chemokines, prostaglandins (PGs), and cytokines, which, in turn, induce the recruitment and activation of immune cells, rapidly triggering an inflammatory response. These pro-inflammatory mediators are sufficient to activate the delivery pathways, resulting in premature uterine activation and contraction, ultimately leading to premature expulsion of the fetus [6,7]. Consequently, some pharmaceutical companies have been focusing on inflammatory pathways as a therapeutic target for preventing and treating PTL.

Proactively delaying the occurrence of PTL post-diagnosis is presently the primary clinical strategy for its prevention and management, with the most prevalent approach involving administering tocolytic agents to inhibit uterine contractions and prolong delivery [8], such as β-agonists (ritodrine and terbutaline), calcium channel blockers (nifedipine), and oxytocin receptor antagonists (atosiban and barusiban) [4,9]. Although calcium channel blockers offer the advantage of an extended gestational duration compared to oxytocin receptor antagonists and β-agonists, all these commonly employed chemical drugs have been associated with a relatively high prevalence of maternal adverse reactions and neonatal morbidity. Some of the primary side effects that have been associated with these medications include maternal chest pains, dyspnea, palpitations, tremors, headaches, hypokalemia, hyperglycemia, nausea/vomiting, and nasal congestion [9,10,11]. Furthermore, although pregnant women with chorioamnionitis are often intervened with antibiotics to prevent and treat infectious PTL, the efficacy of these medications during the middle and late stages of pregnancy remains suboptimal. Additionally, prenatal exposure of infants to antibiotics has been linked to the occurrence of asthma and reduced birth weight [12]. These insights highlight the current lack of safe and efficacious therapeutic approaches to address PTL or improve maternal and neonatal complications associated with premature delivery, posing a major challenge to obstetrics [13].

Natural polysaccharides, extracted from traditional Chinese medicine, have demonstrated various beneficial pharmacological effects on the treatment of human diseases, especially in inflammation-related diseases [14,15,16]. Gfn-type fructooligosaccharides comprise fructose units connected by β-(2→1)- or β-(2→6)-D-glycosidic bonds, with an α-(2→1)-D-glucosyl terminal group [17,18]. Some Gfn-type fructooligosaccharides can reduce pro-inflammatory cytokine production by stimulating Dendritic Cells (DCs) or occupy LPS-bound I124 and other amino acid residues on TLR4, thus alleviating inflammatory responses [19].

Moreover, Gfn-type fructooligosaccharide extracted from burdock (*Arctium lappa* L.) root (BFO) have been confirmed to inhibit the expression of pro-inflammatory cytokines (IL-8, IL-6, IL-1β, and TNF-α) and chemokines (Icam-1, Vcam-1, and MCP-1) in macrophages via downregulation of the TLR4/NF-κB signaling pathway [20]. We previously found that BFO exhibited diverse biological activities in vivo, including anti-inflammatory, immunomodulatory, hypoglycemic, and anti-colitis effects [21,22].

Additionally, other Gfn-type fructooligosaccharides, such as *Atractylodes Macrocephala* polysaccharide (AMP) [23] and *Codonopsis pilosula* polysaccharide (CPP) [24], have also been established to possess immunomodulatory and antioxidant effects. AMP can elevate the levels of secretory immunoglobulin A by inhibiting the TLR4/NF-κB signaling pathway, thereby mitigating the inflammatory response in mice with severe acute pancreatitis [25]. CPP may exert an anti-fibrotic effect on the liver by modulating both the TLR4/NF-κB and TGF-β1/Smad3 signaling pathways [26]. However, the study of the application of fructooligosaccharides on the prevention or treatment of inflammation-related PTL is absent. Herein, we selected BFO, AMP, and CPP as candidates to investigate their efficacies on the preterm labor intervention, and their potential mechanisms to alleviate uterine inflammatory responses and overwhelmed smooth muscle contractions, which may provide valuable insights for developing an innovative drug with superior safety and efficacy for PTL intervention in the future.

## 2. Results

### 2.1. Purification of Fructooligosaccharides

**Figure 1:**
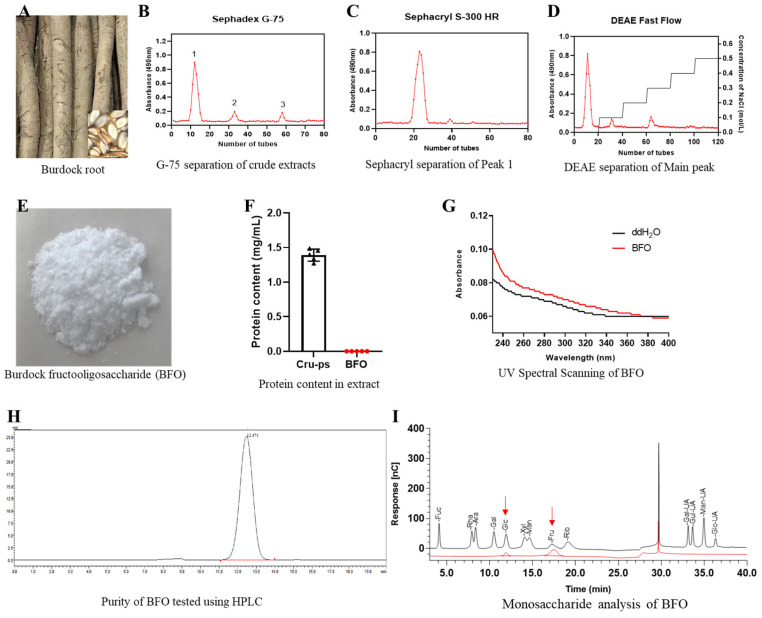
The preparation and identification of fructooligosaccharide. (**A**) Fresh burdock root. (**B**) The elution curve of the separation of crude polysaccharide from burdock root using Sephadex G-75 and followed by further purification of peak 1 utilizing (**C**) Sephacryl S-300 HR and (**D**) DEAE-cellulose-52. (**E**) The lyophilized BFO powder. (**F**) The protein contents in crude polysaccharide (Cru-ps) and BFO were determined. (**G**) UV spectral scanning of BFO and distilled water between 230 and 400 nm was conducted. (**H**) Purity assessment and (**I**) monosaccharide composition analysis were performed for BFO. In the monosaccharide composition analysis, the black line represents the standard chromatogram, while the red line represents the sample chromatogram, and the red arrows indicate the positions of glucose and fructose.

After extracting fresh burdock root (Figure 1A) in hot water at 80 °C, the resulting mixture separated using Sephadex G-75 showed three distinct peaks, with peak 1 being the main peak (Figure 1B). The substance of peak 1 was further purified with Sephacryl S-300 HR (Figure 1C), followed by endotoxin removal via DEAE-cellulose-52 gel chromatography (Figure 1D). Subsequently, the solution at 0 mol/L NaCl was collected and freeze-dried to a white powder: purified BFO (Figure 1E). The protein concentration results demonstrated a higher protein content in the crude extract, whereas no detectable protein was found in the purified BFO (Figure 1F). Additionally, UV scanning of BFO within the 230 nm–400 nm wavelength range revealed no absorption peaks at 260 nm and 280 nm (Figure 1G), indicating that BFO was free of nucleic acids and proteins and was suitable for subsequent administration. The powder’s purity was further determined using HPLC, revealing a purity value of 99.75% (Figure 1H). Furthermore, the monosaccharide composition analysis of BFO revealed that it comprised fructose and glucose units (Figure 1I), confirming that it is a Gfn-type fructooligosaccharide.

The other two Gfn-type fructans, AMP and CPP, were also isolated and purified according to the methods of Wang et al. [27] and Meng et al. [28] for subsequent experiments.

### 2.2. Single Injection of BFO Can Reduce the Occurrence of PTL

Herein, we found that the injection of 50 μg/kg/mouse LPS could stably induce premature birth (occurrence 100%). The duration from LPS injection to the birth of the first pup was approximately 20.5 h, leading to an average gestational period of approximately 16.35 days (Table 1, row 3). If term delivery occured following treatment, the length from LPS injection to the birth of the first pup in the term groups was more than 95 h, and the delivery began on day 19.5 (the lower row of each treatment group).

Notably, treatment with 30 mg/kg/mouse BFO reduced the premature birth rate to 42.86%, and the duration from LPS injection to the birth of the first pup in the context of PTL mice was approximately 20.03 h, which was not significantly different from that in LPS-injection group (Table 1, row 6). A single injection of 60 mg/kg/mouse BFO demonstrated enhanced the efficacy in treating premature birth, reducing the premature birth rate to 28.57% and the premature delivery duration in the context of PTL mice to approximately 28.2 h, which was significantly longer than that of the LPS group (Table 1, row 7). Moreover, a dose of 15 mg/kg of BFO administered to mice had no significant effect on preventing premature birth. However, a single injection of CPP and AMP demonstrated a far more inferior therapeutic efficacy against premature birth relative to BFO at an equivalent concentration (Table 1, row 8 and row 9), making us focus on the BFO treatment in the following studies.

### 2.3. Repeated Injection of BFO Showed Extraordinary Efficacy in Preventing PTL

Based on the preliminary experimental findings presented in Table 1, we explored the therapeutic effects of repeated administration of BFO in premature birth treatment. Specifically, the BFO was administered 2 h and 8 h after LPS injection at the same concentration, which significantly enhanced the efficacy compared to a single administration of BFO in treating LPS-induced PTL (Table 2). At the doses of 30 mg/kg/mouse and 60 mg/kg/mouse BFO, LPS-induced PTL was completely abolished and significantly reduced to 9.09%. The average gestational period was extended to 19.88 ± 0.3 days and 20.2 ± 0.4 days, respectively (Table 2, row 6 and row 7), which is comparable to that in the vehicle control group. Additionally, the survival rate (SR) of pups born in the administration group was comparable to that of control group as well. Consequently, based on the principle of minimal efficient dosages, we ultimately opted to administer two 30 mg/kg/mouse BFO injections at 6 h intervals starting from 2 h after LPS injection as the PTL intervention in subsequent experiments.

### 2.4. BFO Attenuates Inflammation-Induced USMC Contraction and Oxytocin-Induced Uterine Smooth Muscle Strip Contraction In Vitro

**Figure 2:**
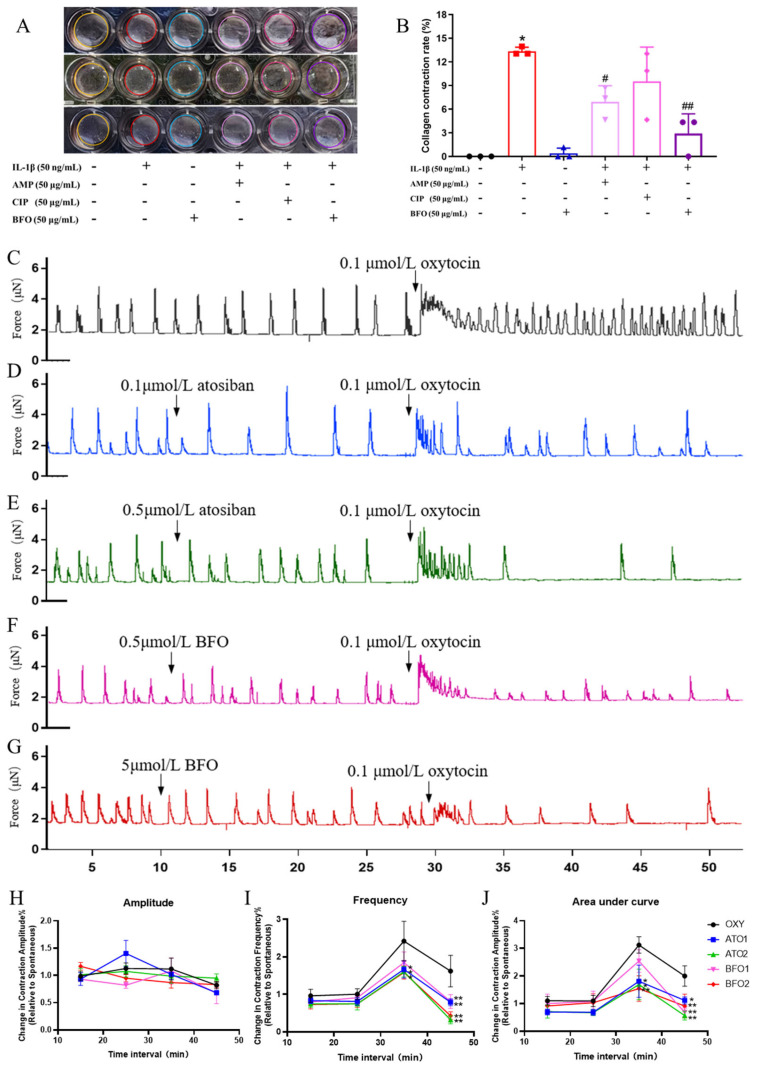
Determination of myometrial cell contractility and the contractile tension of ex vivo myometrial strips. (**A**) Collagen contraction of primary uterine smooth muscle cells was determined and (**B**) their contraction rates were analyzed, the same color circle represents the same treatment replicates. (**C**) The contraction curve of myometrial strips from pregnant mice was induced by 0.1 μmol/L oxytocin in vitro, and the changes in contractions were induced by oxytocin after preconditioning with different drugs including (**D**) 0.1 μmol/L atosiban, (**E**) 0.5 μmol/L atosiban, (**F**) 0.5 μmol/L BFO, and (**G**) 5 μmol/L BFO. The relative changes in the contraction (**H**) amplitude, (**I**) frequency, and (**J**) area under the curve were calculated at intervals of every 10 min compared with the 10 min spontaneous contractions before the administration of the different reagents. * *p* ≤ 0.05 and ** *p* ≤ 0.01 vs. control group; ^#^ *p* ≤ 0.05 and ^##^ *p* ≤ 0.01 vs. LPS group using one-way ANOVA with Tukey’s HSD test. *N* = 3.

Inflammatory cytokine IL-1β significantly induced the contraction of mouse primary uterine smooth muscle cells (USMCs) embedded in collagen (Figure 2A). After adding AMP, CPP, and BFO, the collagen contraction degree was alleviated to varying extents, with reductions observed at approximately 48.21%, 28.66%, and 78.28%, respectively (Figure 2B). Both the AMP and BFO additions manifested significant reductions in USMC contraction compared to the treatment of IL-1β alone, with the BFO addition showing more dramatic efficacy (Figure 2B).

Subsequently, isometric force myography was used to evaluate the impact of BFO on contractions in murine uterine smooth muscle strips in vitro. Isolated uterine smooth muscle strips exhibited spontaneous rhythmic contractions, and the addition of 0.1 μmol/L oxytocin significantly increased the contraction frequency of uterine smooth muscle strips (Figure 2C).

Atosiban is a commonly used oxytocin receptor antagonist to inhibit uterine contractions in the clinic. It can be seen that the pre-treatment of atosiban acetate for 20 min did not affect spontaneous uterine contraction but significantly decreased the contraction amplitude, frequency, and area under the curve (AUC) induced by oxytocin (Figure 2D,E,H,J). The pre-treatment of BFO for 20 min exhibited similar effects on oxytocin-induced contractions. Here, 0.5 μmol/L BFO significantly attenuated the amplitude and AUC of contraction (Figure 2F,H,J), while 5 μmol/L BFO demonstrated an extremely significant decrease in not only the amplitude and AUC but also the frequency of uterine strip contraction induced by 0.1 μmol/L oxytocin, without exhibiting significant effects on spontaneous uterine contraction (Figure 2G,H,J). These findings suggest that BFO might exert similar effects to atosiban to directly suppress oxytocin-induced uterine smooth muscle contractions.

On the contrary, the addition of nifedipine, a widely used calcium channel inhibitor, as a tocolytic agent significantly decreased the amplitude and frequency of the spontaneous contraction of uterine strips while reducing the amplitude but not the frequency of contractions induced by oxytocin, showing a quite different pattern of contraction inhibitory effects from both atosiban and BFO (Figure S1).

### 2.5. In Vivo BFO Is Highly Enriched in Myometrial Tissues but Not in Fetus

**Figure 3:**
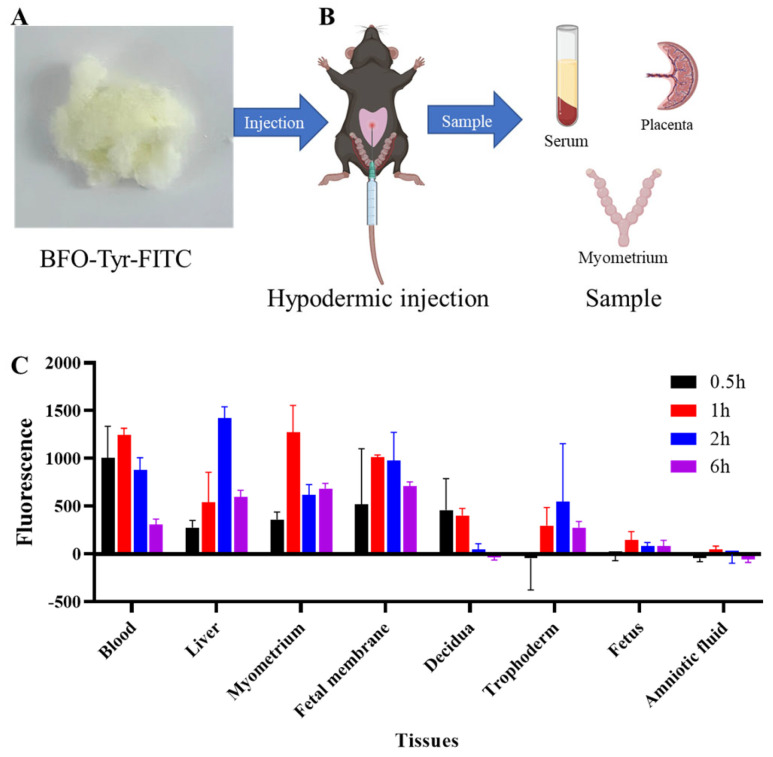
Drug distribution kinetics of BFO in vivo. (**A**) The dry powder of BFO-Tyr-FITC. (**B**) The schematic diagram of BFO-Tyr-FITC injection and tissue collection. (**C**) Histogram of fluorescence intensity changes in the serum, liver, myometrium, fetal membrane, decidua, trophoderm, fetus, and amniotic fluid at 0.5, 1, 2, and 6 h after injection, respectively.

For deeper insights into the therapeutic effects of BFO on LPS-induced PTL and its metabolic distribution in vivo, we subjected it to dynamic distribution studies using fluorescence-labeled BFO (Figure S2). Two hours after administering LPS, BFO-Tyr-FITC was subcutaneously injected into the abdominal region of pregnant mice (Figure 3A). After 0.5 h, 1 h, 2 h, and 6 h of BFO-Tyr-FITC injection, various tissues, including the blood, liver, myometrium, fetal membrane, decidua, trophoderm, fetus, and amniotic fluid, were collected for fluorescence distribution detection (Figure 3B).

The results demonstrated that the fluorescence intensity reached a relatively high level in the maternal blood at 0.5 h after injection and remained stable in the bloodstream within the first 2 h, with a distinct peak observed in the liver at 2 h (Figure 3C). Furthermore, the fluorescence signals were widely distributed in the myometrium, fetal membranes, decidua, and trophoderm, while the fluorescence intensities in the fetus and amniotic fluid were quite low and hard to detect (Figure 3C). Notably, a pronounced enhancement in the fluorescence intensity was observed in the uterine smooth muscle layer tissue, reaching a peak at 1 h post-treatment, indicating that BFO could be delivered successfully to exert its biological effects within this particular tissue.

### 2.6. BFO Is Co-Localized with TLR4 on Uterine Smooth Muscle Cells

**Figure 4:**
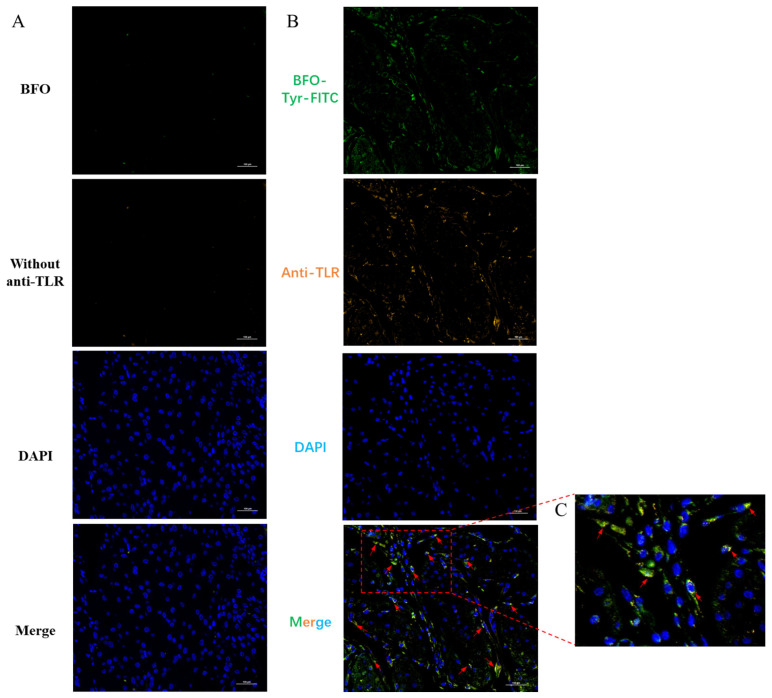
Co-localization of BFO and TLR4 on myometrial cells. The immunofluorescence co-localization between BFO (green) and TLR4 (orange) in mouse myometrium in vivo was determined for (**A**) the control group (without fluorescein) and (**B**) the treatment group (labeled with fluorescein), along with its locally magnified images (**C**). The bright yellow colors pointed by red arrows indicate the co-localization of BFO with TLR4.

Since LPS exerts its effects by binding to toll-like receptor 4 (TLR4) and previous studies have revealed that Gfn-type fructans may demonstrate immune benefits by competitively antagonizing TLR4 [19], we further investigated whether BFO, as a Gfn-type fructooligosaccharide, manifested its anti-inflammatory effects in the myometrium by tethering the TLR4 receptor as well. The immunofluorescence studies demonstrated that 1 h after BFO-Tyr-FITC injection, significant co-localization signals between BFO and TLR4 can be observed on mouse uterine smooth muscle cells (Figure 4A–C), suggesting that BFO may mitigate inflammation-induced preterm labor by interfering with LPS-induced inflammatory responses through tethering LPS receptor TLR4 on uterine smooth muscle cells.

### 2.7. BFO Alleviates the Expression of TLR4-Downstream Genes

**Figure 5:**
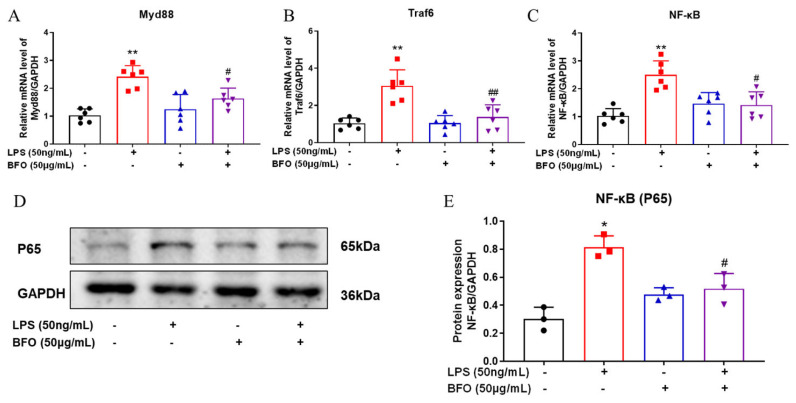
Inhibitory effects of BFO on key downstream genes of TLR4 signaling pathway in the myometrium. The mRNA expression of *MyD88* (**A**), *Traf6* (**B**), and *NF-κB* (**C**) genes, as well as the protein expression of NF-κB subunit P65 (**D**) and its statistical analysis (**E**) in USMCs were presented. The cells were treated with BFO (50 μg/mL) and LPS (50 ng/mL) alone or in combination for 24 h. * *p* ≤ 0.05 and ** *p* ≤ 0.01 vs. control group; ^#^ *p* ≤ 0.05 and ^##^ *p* ≤ 0.01 vs. LPS group using one-way ANOVA with Tukey’s HSD test. *N* ≥ 3.

*MyD88*, *TRAF6*, and *NF-κB* are key downstream genes of the TLR4 signaling pathway, which mediate the inflammatory responses of TLR4 activation. QPCR analyses demonstrated that BFO can significantly downregulate the mRNA expression of the *MyD88*, *TRAF6*, and *NF-κB* genes induced by LPS in the myometrium (Figure 5A–C). Moreover, the LPS-induced protein levels of NF-κB subunit P65 were also reversed by BFO treatment in the myometrium (Figure 5D,E). These findings suggest that BFO may mitigate LPS-induced inflammation by antagonizing TLR4 and blocking its downstream pathway consisting of MyD88, TRAF6, and NF-κB.

### 2.8. BFO Reduces the Elevation of Pro-Inflammatory Cytokines and Chemokines Induced by LPS in Maternal Blood

**Figure 6:**
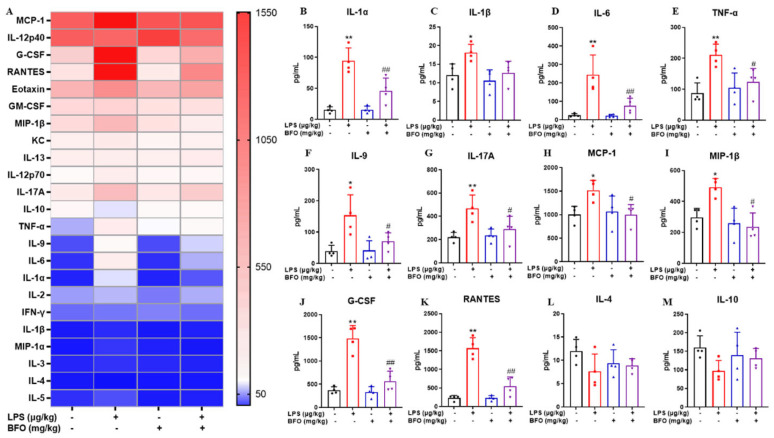
Inflammatory cytokine levels in maternal serum were analyzed using Luminex technology. (**A**) Heat map results of inflammatory cytokines in the sera of pregnant mice were obtained after 19 h of LPS and/or BFO injection. Significant differences were observed in pro-inflammatory and chemotactic cytokines, including (**B**) IL-1α, (**C**) IL-1β, (**D**) IL-6, (**E**) IL-9, (**F**) IL-12p70, (**G**) IL-17A, (**H**) MCP-1, (**I**) MIP-1β, (**J**) G-CSF, and (**K**) RANTES, as well as levels of anti-inflammatory cytokines (**L**) IL-4 (**M**) and IL-10 among different groups. * *p* ≤ 0.05 and ** *p* ≤ 0.01 vs. control group; ^#^ *p* ≤ 0.05 and ^##^ *p* ≤ 0.01 vs. LPS group using one-way ANOVA with Tukey’s HSD test. *N* = 4.

Since LPS treatment was known to induce an apparent pro-inflammatory response systemically, we investigated if BFO administration could counteract the pro-inflammatory effects of LPS. Maternal serum samples were collected at 19 h post-LPS injection, and the expression levels of inflammatory cytokines and chemokines were determined using Luminex technology (Figure 6A). It can be seen that the levels of pro-inflammatory cytokines (IL-1α, IL-1β, IL-6, TNF-α, IL-9, and IL-17A) and chemokines (MCP-1, MIP-1β, G-CSF, and RANTES) were significantly increased in the serum upon the treatment of LPS compared to the control group (Figure 6B–K). However, BFO pre-treatment effectively suppressed the upregulation of these inflammation-related cytokines and chemokines to varying extents, particularly exhibiting significant reversals of IL-1α, IL-6, TNF-α, MCP-1, MIP-1β, G-CSF, and RANTES expressions, compared to that in the LPS group (Figure 6B–K). In addition, although no statistically significant differences were shown in the bar graphs, the anti-inflammatory cytokines IL-4 and IL-10 exhibited decreasing trends upon LPS treatment, which were slightly reversed by the pre-treatment of BFO as well (Figure 6L,M). Notably, BFO pre-treatment alone exerted minimal impact on inflammatory cytokine and chemokine expression; hence, we postulated that BFO may specifically mitigate the LPS-induced pro-inflammatory responses in pregnant mice without disturbing immune homeostasis, potentially contributing to its therapeutic efficacy and safety against PTL.

### 2.9. BFO Alleviates LPS-Induced Changes in Progesterone and Estradiol in Maternal Blood

**Figure 7:**
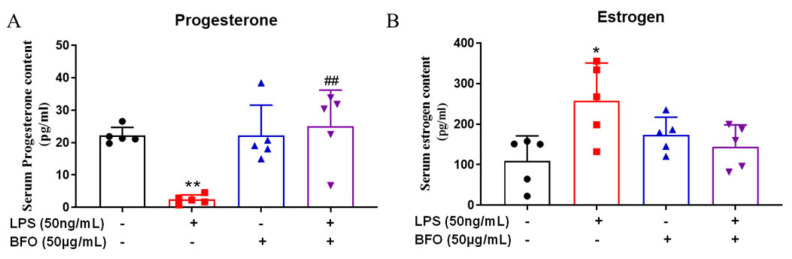
Progesterone and estradiol levels in maternal serum were analyzed using ELISA kits. The changes in the (**A**) progesterone content and (**B**) estradiol content in the sera of pregnant mice were obtained after 19 h of LPS and/or BFO injection. * *p* ≤ 0.05; ** *p* ≤ 0.01 vs. control group; ^##^ *p* ≤ 0.01 vs. LPS group using one-way ANOVA with Tukey’s HSD test. *N* = 4.

Progesterone and estradiol can regulate the immune status of the uterus. The ELISA results demonstrated that compared with the control group, LPS induced an extremely significant decrease in progesterone levels (Figure 7A) and a significant increase in estradiol levels (Figure 7B). The administration of BFO mitigated these changes, restoring hormone levels closer to those observed in the control group (Figure 7A,B). These significant changes in the progesterone and estradiol levels are closely associated with the occurrence of PTL, which further supports the potential of BFO to reduce the incidence of PTL.

### 2.10. BFO Reduces LPS-Induced Inflammatory Cytokine Expression in the Myometrium

**Figure 8:**
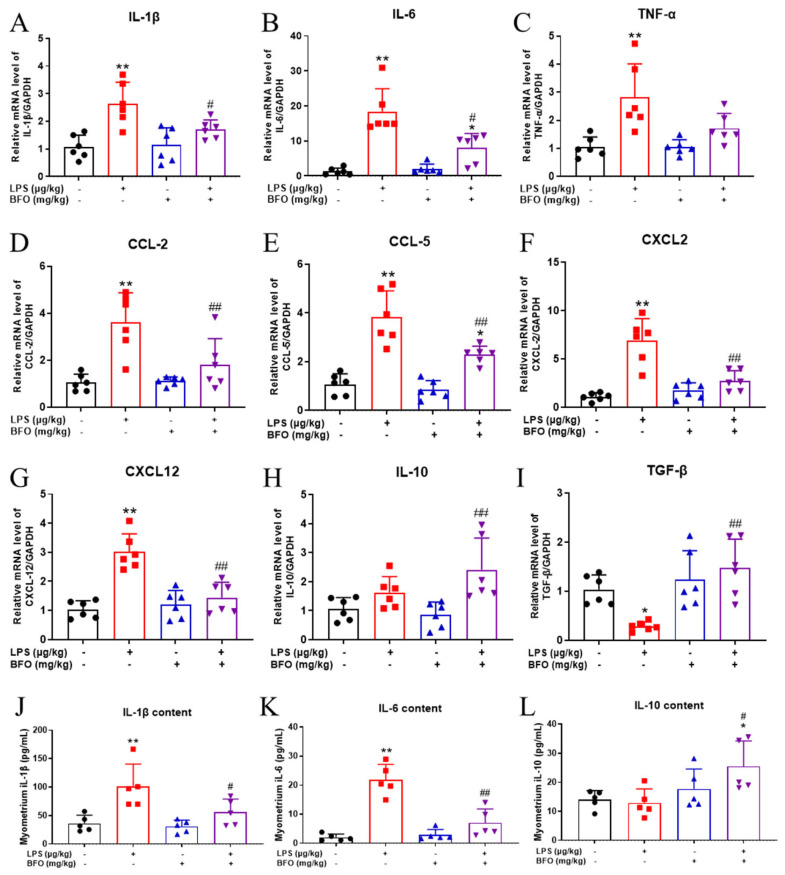
The mRNA and protein expressions of inflammatory cytokines in the myometrium. The mRNA expression levels of inflammation-related cytokines in the myometrium, including (**A**) IL-1β, (**B**) IL-6, (**C**) TNF-α, (**D**) CCL-2, (**E**) CCL-5, (**F**) CXCL2, (**G**) CXCL12, (**H**) IL-10, and (**I**) TGF-β were examined. And changes in the content of inflammatory cytokines, including (**J**) IL-1β, (**K**) IL-6, and (**L**) IL-10, in the myometrium were also analyzed. * *p* ≤ 0.05 and ** *p* ≤ 0.01 vs. control group; ^#^ *p* ≤ 0.05 and ^##^*p* ≤ 0.01 vs. LPS group using one-way ANOVA with Tukey’s HSD test. *N* ≥ 5.

To further determine if BFO can also counteract the pro-inflammatory responses induced by LPS in the myometrium locally, the expression of inflammation-related cytokines and chemokines was analyzed using real-time qPCR. Compared to the vehicle control group, the mRNA expressions of pro-inflammatory cytokines (IL-1β, IL-6, and TNF-α) and chemokines (CCL2, CCL5, CXCL2, and CXCL12) in the myometrium were significantly upregulated by the LPS treatment (Figure 8A–G), which is largely consistent with the phenotype observed in the maternal serum. Similarly, BFO pre-treatment alleviated the upregulation of these cytokines to varying extents, with the reversals of IL-1β, IL-6, CCL2, CCL5, CXCL2 and CXCL12 reaching statistical significance (Figure 8A,B,D–G). Although the expression of the anti-inflammatory cytokine IL-10 was not significantly downregulated upon LPS treatment, the pre-treatment of BFO significantly increased the expression of IL-10 in the myometrium compared to LPS treatment alone (Figure 8H).

To determine the protein levels of the inflammatory cytokines, we next performed inflammatory cytokine quantification in myometrial tissues using the ELISA method, revealing a trend comparable to that of the serum. Consistent with the mRNA expression results, BFO pre-treatment significantly attenuated the LPS-induced increase in IL-1β and IL-6, with reductions of approximate 44.74% and 67.81%, respectively (Figure 8I,J). Similar to the mRNA expression, the protein expression of IL-10 was also not significantly decreased by LPS treatment alone but was significantly increased upon BFO pre-treatment (Figure 8K), exhibiting an approximately double increase compared to that observed in the LPS group.

### 2.11. BFO Reduces LPS-Induced Inflammatory Cells Infiltration into the Myometrium

**Figure 9:**
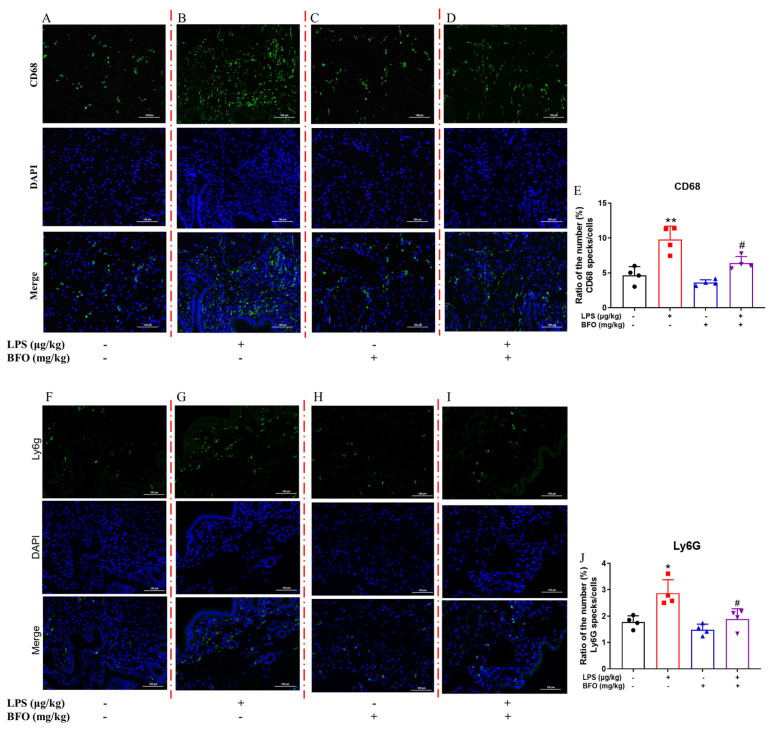
The infiltration of inflammatory cells into the myometrium. Macrophage infiltration was photographed at 40× magnification in the (**A**) control group, (**B**) LPS administration group, (**C**) double dose of BFO administration group, and (**D**) LPS combined with double doses of BFO administration group; (**E**) their positive staining rate statistics. Neutrophil infiltration was similarly imaged at 40× magnification in the (**F**) control group, (**G**) LPS administration group, (**H**) double dose of BFO administration group, and (**I**) LPS combined with double doses of BFO administration group; (**J**) their positive staining rate statistics. Red dashed lines are used to distinguish immunofluorescence staining for different treatments, with green representing target protein fluorescence and blue representing DAPI staining. Scale bar: 100 μm. * *p* ≤ 0.05 and ** *p* ≤ 0.01 vs. control group; ^#^ *p* ≤ 0.05 vs. LPS group using one-way ANOVA with Tukey’s HSD test. *N* = 4.

Provided that chemokines, such as CCL2, CCL5, CXCL2, and CXCL12, induced by LPS were significantly reversed by BFO in the myometrium, we further verified the effect of BFO on immune cell chemotaxis in the myometria of PTL mice using immunofluorescence staining. It was found that macrophage infiltration was significantly increased in the myometrium within 19 h after LPS injection compared to the vehicle control group (Figure 9A,B), whereas the administration of BFO effectively mitigated macrophage infiltration by approximately 34.46%, compared to that in the LPS group (Figure 9C,D), to an extent comparable to the control group (Figure 9E). In addition, ly6g-labeled neutrophils showed a similar trend (Figure 9F–J). These findings suggest that BFO may attenuate the LPS-induced heightened macrophage chemotaxis in the myometrium, highlighting its potential significance in PTL treatment by blocking the overwhelmed inflammatory activation.

## 3. Discussion

Although several PTL management strategies have already been implemented in some developed or developing countries, effective and safe treatment options remain scarce owing to the complex etiology and complications [29,30]. Given the severe drug side effects, most chemicals currently used to address PTL often result in significant neonatal morbidity and maternal adverse reactions [9]. Natural polysaccharides can modulate various immune functions through multiple receptors and signaling pathways (such as TLRs, MAPK, or NF-κB) [14,31]. Fructooligosaccharides, a subclass of natural polysaccharides primarily comprising multiple fructose units, can mitigate inflammation, exert antiviral effects, enhance calcium absorption, and protect against oxidative stress [32,33]. Since PTL has often been associated with the dysregulation of immune functions and inflammation processes, it is worthwhile to investigate the potential protective effects of natural polysaccharides in the occurrence of PTL. Herein, different types of Gfn-type fructooligosaccharides, including BFO, AMP, and CPP, which had been reported to improve mice immunological functions and alleviate vascular inflammation [21,34], were employed to explore their protective effects on inflammation-induced PTL. Among them, only the single dose of BFO can effectively mitigate LPS-induced PTL, which resulted in almost complete remission upon repeated administration of 30 mg/kg/mouse BFO, without any apparent increase in neonatal morbidity and mortality. A similar ameliorative effect was observed in mouse uterine smooth muscle cell contraction experiments, with BFO being the most effective agent.

Atosiban acetate and nifedipine can significantly attenuate the oxytocin-induced contraction of uterine smooth muscle strips, but the inhibitory patterns were significantly different. Atosiban is an oxytocin receptor antagonist [35], and nifedipine is a calcium channel blocker [36]. Interestingly, BFO demonstrated a similar inhibitory effect to atosiban. It did not affect the spontaneous contraction of smooth muscle strips but significantly decreased the frequency of contraction while having mild effects on the amplitude of contraction, naturally making us to speculate that BFO may possess the ability to antagonize oxytocin receptors to inhibit myometrial contractions. However, the interference of BFO on the association between oxytocin receptors and TLR4 receptors may be a critical and in-depth focus in our subsequent research, revealing its clinical application value.

Inflammation is a critical component of the immune system, with its primary function being to eliminate microbial invasions. Toll-like receptors (TLRs) recognize a diverse array of pathogen-associated molecular patterns, playing a pivotal role in inflammatory regulation and immune responses [37]. Numerous studies have reported that uterine inflammatory cascade responses were activated before labor and PTL in rodents and humans, often initiated by the activation of toll-like receptor 4 (TLR4). This process primarily involves the release of inflammatory cytokines and subsequent leukocytes infiltration into the maternal–fetal interface, cervix, and myometrium, which will stimulate a synchronous increase in the expression of contractile proteins within the myometrium to facilitate uterine contractions. These inflammatory cascades could further promote fetal membrane rupture and cervical ripening, ultimately causing parturition [5,38,39]. In our inflammation-induced preterm labor model, LPS is a primary agonist of TLR4, and its interaction with this receptor leads to the production of pro-inflammatory cytokines, as well as platelet activating factor, prostaglandins, and nitrogen species [40].

Interestingly, dynamic distribution analysis revealed a significant enrichment of BFO in the uterine smooth muscle layer, as well as its perfect co-localization with LPS receptor TLR4 on mouse uterine smooth muscle cells. These findings together indicated that BFO was successfully delivered to this specific tissue, thereby binding to TLR4 to antagonize LPS-induced inflammatory processes, including inflammatory signaling molecules and subsequent chemokines/cytokines releases, ultimately exerting its tocolytic effects, which are consistent with the previous findings of Fernández-Lainez et al. [19] and Zeng et al. [20].

Previous studies have reported that polysaccharides derived from *Arctium lappa* L. could exert inhibitory effects on pro-inflammatory cytokines, intercellular adhesion molecule-1 (Icam-1), vascular cell adhesion molecule-1 (Vcam-1), and monocyte chemoattractant protein-1 (MCP-1) [18], most of which are closely associated with the occurrence of PTL [41,42]. Consistently, we found that BFO administration significantly reversed the increases in numerous pro-inflammatory cytokines and chemokines induced by LPS in either maternal serum or its target organ myometrium, thereby mitigating the occurrence of PTL caused by inflammatory cascade reactions. Notably, some existing drugs used for treating and preventing preterm birth, such as glucocorticoids [43] and magnesium sulfate [44], can potentially affect fetal development by crossing the placental barrier. However, in our study, BFO administration alone neither crossed the placental barrier to affect the normal development of fetus nor disturbed basal immune homeostasis at the maternal–fetal interface, once again highlighting that it is a rather safe therapeutic tocolytic agent for PTL prevention, with minimal side effects for both the mother and fetus.

It is well known that estrogen (E2) and progesterone (P4) play crucial roles in pregnancy, particularly in regulating the quiescent state of the uterine myometrium [45]. Functional withdrawal of P4 is regarded as an important trigger of parturition by the diminished effects on maintaining the quiescence of the uterine myometrium [46]. Consistent with our findings, LPS administration resulted in an extremely significant decrease in serum P4 in mice, potentially compromising the maintenance of uterine myometrial quiescence. Estrogen has been demonstrated to upregulate the expression of contractile-related proteins, including cyclooxygenase-2 (COX2), connexin 43 (Cx43), and oxytocin receptor (OXTR). Additionally, estrogen plays a crucial role in mediating the inflammatory response, increases the production of pro-inflammatory cytokines, which is an essential component of the parturition process [45,47]. Our results showed that the administration of LPS significantly increased the estradiol levels in mouse serum, which may contribute to increased pro-inflammatory cytokine production and myometrial contractions. Interestingly, BFO treatment could restore the levels of both hormones to levels comparable to those of the control group, further suggesting the potential role of BFO in preventing PTL. However, the interaction between the changes in E2 and P4 and the antagonistic effects of BFO on the LPS/TLR4 complex warrant further investigation in a future study.

Various inflammatory cells, predominantly macrophages and neutrophils, could be attracted by chemokines and infiltrate the myometrium, leading to the release of pro-inflammatory cytokines such as IL-1β, TNF-α, and IL-8, reciprocally worsening the inflammatory microenvironment and expediting labor onset [48,49]. As expected, LPS induced the significantly increased infiltration of leukocytes into the myometrium, a phenomenon that closely correlates with PTL occurrence [49,50]. Notably, BFO administration significantly alleviated the infiltration of macrophages and neutrophils in the myometrium, which is consistent with the reversed upregulation of chemokines such as CCL2, CCL5, CXCL2, and CXCL12 by BFO. Studies have demonstrated significant upregulation of these chemokines in aborted or preterm mouse uteruses [51,52], highlighting their potential involvement in host defense mechanisms against bacterial infections and chemotactic responses [53]. The efficacy of BFO in attenuating these chemokines and inflammatory cells infiltration once again underscores its potential in resisting inflammation-induced PTL.

There are still several limitations of the current study. Firstly, we observed the preventive effects of BFO on preterm labor in a mouse model. Due to the ethical limitation, it is still a long way from applying this treatment to humans. However, regarding the highly conserved structure of TLR4 between mice and humans, it is speculated that BFO will be playing a similar role in the prevention of human PTL. Secondly, we mainly focused on the efficacy of BFO in treating PTL without dissecting the in-depth molecular mechanisms. In a future study, we will conduct RNA-sequencing analysis and further investigate the possible molecular mechanisms underlying the competitive inhibition of TLR4 by BFO.

## 4. Materials and Methods

### 4.1. Isolation and Purification of Fructooligosaccharide

The extraction process mainly refers to the method slightly modified from Ma et al. [24] and Wang et al. [54]. Fresh burdock roots were sliced into pieces, extracted with 80 °C distilled water three times, and filtered to collect the immersion solution. The immersion solutions were concentrated to 1/4 of the original volume, and then ethanol (Macklin Biochemical Technology Co., Ltd., Shanghai, China) was added to obtain a final concentration of 75%. The mixture was incubated overnight at 4 °C to precipitate the crude polysaccharides. The crude polysaccharides were dissolved with distilled water, and the proteins were removed using the Sevage method. The sample was decolorized with the macroporous, weakly basic anion-exchange resin D301R (Guangfu Technology Development Co., Ltd., Tianjin, China), and the components were separated through Sephadex G-75 (Yuanye Biotechnology Co., Ltd., Shanghai, China) and Sephacryl S-300 HR (Yuanye Biotechnology Co., Ltd., Shanghai, China) gel chromatography columns, followed by purification with a DEAE-cellulose-52 (Yuanye Biotechnology Co., Ltd., Shanghai, China) exchange column to obtain a single component, BFO.

The *Atractylodes macrocephala* polysaccharide (AMP) was isolated and purified following the method of Wang et al. [27]. Briefly, the rhizome of *Atractylodes macrocephala* was dried at 40 °C and ground into a powder. The rhizome powder was extracted with distilled water at a ratio of 1:20 (*w*/*v*) at 80 °C for 4 h. Ultrasound-assisted extraction was performed 3 times, each time for 30 min. The collected solution was mixed with hydrogen peroxide at 37 °C. S proteins were removed using the Sevage reagent. Ethanol was added to achieve a final concentration of 75% and kept at 4 °C for 48 h to precipitate crude polysaccharides. The crude polysaccharide was separated and purified through DEAE-52-cellulose (Yuanye Biotechnology Co., Ltd., Shanghai, China) and Sephadex G-100 (Yuanye Biotechnology Co., Ltd., Shanghai, China) columns to obtain AMP.

The *Codonopsis pilosula* polysaccharide (CPP) was isolated and purified according to the method described by Meng et al. [28]. Briefly, crude powders of *Codonopsis pilosula* were added into petroleum ether, after reflux extraction for 1 h. The resulting residues were then soaked overnight in 80% ethanol, followed by a second reflux extraction for 1 h. Subsequently, the residues were extracted with water through reflux for 1.5 h. The supernatant was deproteinized using the Sevage method. Ethanol was added to achieve a final concentration of 75%, and the mixture was incubated overnight at 4 °C to precipitate the crude polysaccharide. The crude polysaccharide was further purified via sequential separation through DEAE-52 cellulose and Sephadex G-100 columns (Yuanye Biotechnology Co., Ltd., Shanghai, China) to obtain CPP.

### 4.2. Identification of BFO

The protein contents of the crude polysaccharide and BFO were analyzed using a BCA rapid protein assay kit (Beyotime, Shanghai, China). The purified BFO was subjected to UV scanning within the wavelength range of 230 nm–400 nm on a microplate reader (Epoch 2, Bio Tek Instruments, South Burlington, VT, USA) to analyze peak changes at 260 nm and 280 nm. The purity assay was conducted using a Shimadzu HPLC system (Shimadzu, Kyoto, Japan) equipped with G3000PWXL chromatographic columns (7.8 × 300 mm, TSK gel, Tosoh Corporation, Yamaguchi Prefecture, Japan) and an RID detector at 40 °C. Ultra-pure water was employed as the mobile phase, while the flow rate was set at 0.7 mL/min. The monosaccharide composition analysis was performed using an ion chromatograph (ICS5000, Thermo Fisher, Waltham, MA, USA) with a Dionex CarbopacTMPA20 column (3 × 150, Dionex, Los Angeles, CA, USA) and an electrochemical detector at 30 °C [22].

### 4.3. Preterm Birth Labor Animal Model

In the present study, we established a mouse model of preterm birth induced by Lipopolysaccharide (LPS) (Escherichia coli 0111:B4, Sigma, St. Louis, MO, USA), following the method described in a previous study [55], with appropriate modifications. C57BL/6J mice were purchased from Jiangsu Huachuang Sino Medical Technology Co., Ltd. (Taizhou, China). Mice aged 8 weeks and weighing 18 ± 2 g were acclimated for one week at a temperature of 20–25 °C and humidity of 50 ± 5%. The mice were then mated overnight with a male-to-female ratio of 1:2, and vaginal plugs were examined in female mice at 8:00 am on the following day to confirm successful mating, which was regarded as day 0.5 of pregnancy (D0.5).

**Figure 10:**
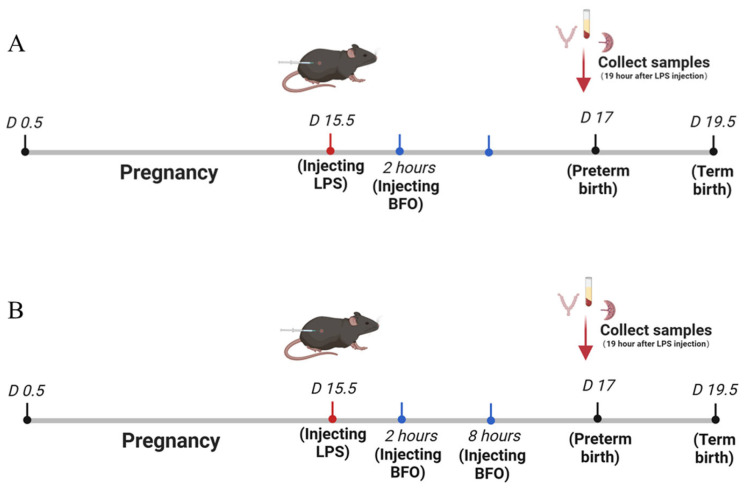
The scheme of establishing an animal model for preterm labor and the single injection (**A**) and double injection (**B**) of BFO treatment.

The LPS was subcutaneously injected into the abdominal region of pregnant mice on day 15.5 of gestation (D15.5). Considering the birth of the first pup as a reference point, offsprings delivered before 19.5 days of gestation were classified as premature. The injection concentration and dosage of LPS were explored to establish a stable mouse model of inflammation-induced preterm labor (Figure 10), and finally, a dose of 50 μg/kg mouse LPS was selected as the optimal dose for inducing preterm labor.

### 4.4. Administration of BFO to Preterm Labor Mice

The experiment was conducted in two series: (a) The first was the single-dose BFO treatment group, where different doses of BFO solution (15, 30, and 60 mg/kg mouse) were administered subcutaneously to mice 2 h after LPS administration at D15.5 (Figure 10A). The dosage concentration was primarily referenced in the method of Li, et al. [56], with appropriate modifications. Additionally, 60 mg/kg AMP and 60 mg/kg CPP were also administered subcutaneously under the same conditions. Each group contained 7 mice, resulting in a total of 56 mice. (b) The second was the double-dose BFO treatment group, where different doses of BFO solution were subcutaneously injected at 2 h and 8 h after LPS administration at D15.5, with the same doses for both injections (Figure 10B). Each group contained 11 mice, resulting in a total of 66 mice. By analyzing the preterm birth rate, the time to deliver the first pup after LPS injection, the average gestation length distinguishing between full-term and preterm labor, and the pup survival rate at term birth, we determined the optimal administration concentration of BFO to clarify its effect on preventing inflammation-induced preterm labor.

### 4.5. Isolation and Culture of Primary Uterine Smooth Muscle Cells

For the method for isolating and culturing primary uterine smooth muscle cells (USMCs), we referred to that of Chao et al. [51], with slight modifications. Simply, the pregnant mouse uterus was collected and cut into small pieces at 4 °C. Subsequently, the tissue was washed three times with pre-cooled D-Hank’s buffer using a vortex mixer and then immersed in an equal volume of DMEM–high-glucose medium (Gibco, Grand Island, NY, USA) supplemented with 0.2% type II collagenase (Gibco, Grand Island, NY, USA), 0.05% Dnase (Beyotime Biotechnology, Shanghai, China), and 0.1% BSA (Biosharp, Hefei*,* China). The mixture was then incubated at 37 °C in a water bath with shaking at 160 rpm for 45 min, followed by adding twice the volume of DMEM complete medium to terminate the digestion reaction. The DMEM complete medium (Gibco, Grand Island, NY, USA) contained fetal bovine serum (ExCellBio, Shanghai, China) at a concentration of 10% *v*/*v* and Penicillin–Streptomycin liquid (Gibco, Grand Island, NY, USA) at a concentration of 1% *v*/*v*. The resulting solution was filtered through a cell filter with a pore size of 37 μm and centrifuged at 1000 rpm to obtain the single-cell suspension. If there are still large residual tissues after filtration, secondary digestion can be performed by adding 1/3 of the volume of digestive solution.

### 4.6. Collagen Contraction Experiment of Uterine Smooth Muscle Cells

In total, 400 μL of digested primary uterine smooth muscle cell suspension at a concentration of 10^5^ cells/mL was mixed with 175 uL of type I collagen (rat tail, Corning Incorporated, Corning, NY, USA) to achieve a final collagen concentration of 1 mg/mL. Then, 4 μL of 1 mol/L NaOH was added to adjust the pH to neutral. The pre-mixed solution turned light red in color and was promptly transferred into a 24-well plate for gelation at 37 °C for 60 min. Then, 500 μL of DMEM–high-glucose medium (Gibco, Grand Island, NY, USA) containing 50 ng/mL IL-1β (MCE, Kenilworth, NJ, USA), 50 μg/mL BFO, 50 μg/mL AMP, or 50 μg/mL CPP was added into different wells alone or in combination. The plates were monitored and photographed every 6 h to calculate the contraction rate using the following formula:

Contraction rate (%) = (experimental collagen diameter − control collagen average diameter)/control collagen average diameter × 100.(1)

### 4.7. Detection of Contractility of Isolated Uterine Smooth Muscle Strips

The myometrial tissues were collected from mice euthanized on the 19th day of pregnancy and immediately transferred to a modified physiological salt solution (PSS) buffer at 4 °C. The PSS buffer consists of the following reagents at the indicated concentrations (mmol/L): 119 NaCl, 4.7 KCl, 1.2 MgSO_4_, 2.0 CaCl_2_, 23 NaHCO_3_, 10.5 glucose, 0.026 EDTA, and 1.2 KH_2_PO_4_. Additionally, fresh CaCl_2_ and glucose were added prior to each use. The uterus was dissected into equal-length longitudinal strips (3–4 mm × 6 mm) and mounted on two pins of an isometric myograph (620 M, DMT, Denmark). It was then immersed in a bath of 5 mL PSS buffer filled with 95% O_2_–5% CO_2_ at 37 °C. All test strips were stretched to a passive tension of approximately 1.5 mN and allowed the baseline to stabilize for 30 min. Various reagents, including 0.1 μmol/L or 0.5 μmol/L atosiban acetate (MCE, Kenilworth, NJ, USA) and 0.5 μmol/L or 5 μmol/L BFO, were introduced approximately 20 min before adding oxytocin to record the changes in myometrial contractions. The relative changes in the contraction amplitude, frequency, and area under the curve were calculated at intervals of every 10 min compared with the 10 min spontaneous contractions before the administration of the different reagents. We recorded the data twice, with a 1 min interval after adding oxytocin.

### 4.8. Construction of Fluorescein-Labeled BFO

BFO-Tyr-FITC was prepared as previously described Xu et al. [57]. Briefly, 100 mg BFO was dissolved into 45 mL of PBS (0.2 mol/L, pH 8.0) and mixed with 80 mg tyramine (Tyr) for a reaction period of 24 h. Following that, 25 mg of sodium borohydride was added, and the mixture was incubated at 37 °C with shaking for 96 h. The supernatant was subsequently purified using a Sephadex G50 column (Yuanye Biotechnology Co., Ltd., Shanghai, China). The eluents containing BFO-Tyr were collected, dialyzed, and lyophilized. The sample was scanned via ultraviolet spectral scanning at wavelengths ranging from 230 nm to 400 nm to verify the success of the amination reaction.

Subsequently, BFO-Tyr was dissolved into water, and the pH was adjusted to 8.5 using 0.2 mol/L sodium carbonate. Then, 5 mg FITC (MCE, Kenilworth, NJ, USA), which had been dissolved in methanol, was added and shaken for 12 h in the dark. The sample was purified via alcohol precipitation followed by elution using Sephacryl S300 HR column chromatography. The eluents containing BFO-Tyr-FITC were collected based on the fluorescence intensity, dialyzed, and lyophilized. The resulting powders were analyzed via infrared scanning and compared with the infrared spectrum of BFO to ascertain whether there were any significant alterations in its main functional groups subsequent to FITC labeling.

### 4.9. Tissue Distribution Analysis of BFO

BFO-Tyr-FITC was subcutaneously injected into pregnant mice at D19, two hours after LPS administration. Then, pregnant mice with or without BFO-Tyr-FITC injection were anesthetized and sacrificed. Their blood, livers, uterine muscles, fetal membranes, decidua, trophoblasts, fetuses, and amniotic fluid were collected at 0, 0.5, 1, 2, and 6 h post-BFO-Tyr-FITC injection following the procedures described by Zhang et al. [58]. An equal volume of PBS (pH 7.4) was added to each tissue sample for homogenization before centrifugation at 7500 rpm for 15 min. The supernatants were collected and mixed with a two-fold saturated ammonium sulfate ((NH4)_2_SO_4_), thoroughly shaken, and allowed to precipitate at room temperature for six hours. After centrifugation at 7500 rpm for 15 min, the supernatants were subjected to fluorescence detection with an excitation wavelength of 490 nm and an emission wavelength of 530 nm. Mice were injected with an equal volume of saline after LPS administration as the control, and the changes in fluorescence intensity of different tissues were calculated.

### 4.10. Immunofluorescence Co-Localization of BFO and TLR4 on USMC

The myometrial tissue of pregnant mice at D19 after injection of fluorescently labeled BFO-Tyr-FITC for 1 h was sampled to determine the immunofluorescence co-localization between BFO and TLR4. Primary antibodies for TLR4 (product code: GB15186) was purchased from Servicebio Biotechnology Co., Ltd. (Wuhan, China). The method was referenced from that of Xu et al. [57]. Simply, the tissue was fixed in 4% paraformaldehyde, followed by dehydration through a graded ethanol series and clarification with xylene. It was subsequently embedded in paraffin and processed into paraffin sections. The sections were then deparaffinized using a combination of xylene and a graded ethanol series. Antigen retrieval was performed using citric acid buffer. After overnight incubation with anti-TLR4 antibody at a dilution of 1:200, it was incubated with a rabbit recombinant fluorescein secondary antibody (product code: GB28301, Servicebio Biotechnology Co., Ltd., Wuhan, China) at a dilution of 1:500 in the dark for 1 h and then stained with DAPI (Beyotime Biotechnology Co., Ltd., Shanghai, China) for 5 min. Finally, tissue fluorescence was determined using a fluorescence inverted microscope (Nikon, Tokyo, Japan).

### 4.11. Expression of Key Genes Downstream of TLR4

Two hours after the LPS injection in pregnant mice at 15.5 days of gestation, BFO was administered at a dose of 30 mg/kg for 6 h, followed by the myometrial tissue being sampled to determine the mRNA expression of *MyD88*, *Traf6*, and *NF-κB* in TLR4/NF-κB pathway by qPCR. And NF-κB proteins were analyzed using Western blotting.

### 4.12. RNA Extraction and Quantitative Real-Time PCR

Total RNAs were extracted from myometrial tissues using TRIzol reagent (Invitrogen, Carlsbad, CA, USA), and cDNA was synthesized from total RNA with a PrimeScript™ RT reagent Kit (TaKaRa, Kyoto, Japan). Real-time PCR was performed on the CFX Duet fluorescence quantitative PCR Instrument (Bio-Rad, Hercules, CA, USA) with Thunderbird SYBR qPCR Mix (Toyobo, Osaka, Japan), according to the manufacturer’s instructions. The relative expression levels of the target genes were calculated using the 2^−△△Ct^ formula, with GAPDH as the housekeeping gene. Each experiment was repeated at least four times. The primer pairs used are listed in Table S1.

### 4.13. Western Blot

The myometrial tissues (20 mg) were homogenized using a multi-sample freeze grinder (Wonbio Biotechnology Co., Ltd., Shanghai, China) with 400 μL of protein lysis buffer. The protein content was measured using the BCA protein quantitative kit (Beyotime, Shanghai, China). The proteins were mixed with loading buffer and heated at 95 °C for 10 min. Twenty micrograms of protein were loaded into a 10% SDS-PAGE gel and transferred onto a PVDF membranes in an ice bath. The membranes were blocked with skim milk and incubated with primary antibodies against the NF-κB p65 subunit (D14E12, Cell signaling technology, Danvers, MA, USA) or GAPDH (ab181602, Abcam, Cambridge, UK) at a dilution of 1:10,000 overnight at 4 °C. After rinsing three times with TBST, the membranes were incubated with secondary antibodies and visualized using the Tanon-5200 multi-functional imaging system (Tanon, Shanghai, China). The densities of the bands representing the p65 subunit or GAPDH (internal control) were analyzed using Image J 1.49V software (National Institutes of Health).

### 4.14. Cytokine Assays in Serum and the Myometrium

Inflammatory cytokine and chemokine concentrations in serum were determined 19 h after LPS injection using Luminex technology with a mouse 23-multiplex cytokine assay on a Bio-Plex^®^ MAGPIX™ Multiplex Reader and Bio-Plex MAGPIX System (Wayen Biotechnologies Inc., Shanghai, China). Changes in the cytokines (IL-1β, IL-6, and IL10) in the mouse myometrium were measured using Enzyme-Linked Immunosorbent Assay (ELISA) kits (YObibio Biotechnology Co., Ltd., Shanghai, China).

### 4.15. Progesterone and Estradiol Assays in Serum

Changes in progesterone and estradiol in mouse serum were determined using the mouse PROG ELISA kit (F11570, Westang Biotechnology Co., Ltd., Shanghai, China) and mouse E2 ELISA kit (F10440, Westang Biotechnology Co., Ltd., Shanghai, China).

### 4.16. Infiltration Analysis of Inflammatory Cells

The fresh myometrial tissues from each group were sampled 19 h after LPS injection, fixed with 4% paraformaldehyde for 60 min, and then washed three times for 5 min with 0.1% PBS-T (Servicebio Biotechnology Co., Ltd., Wuhan, China). Non-specific binding was blocked with a 5% BSA solution at room temperature for 1 h. Then, the tissue slices were incubated with the CD68+ and Ly6g antibodies overnight at 4 °C. Primary antibodies for CD68+ (product code: GB115723) and Ly6g (product code: GB11229) were purchased from Servicebio Biotechnology Co., Ltd. (Wuhan, China). And then the second antibody (Alexa Fluor 488, product code: GB28303, Servicebio Biotechnology Co., Ltd., Wuhan, China) was diluted in a 5% BSA solution at a ratio of 1:200, followed by incubation at room temperature for 1 h. Subsequently, after rinsing with PBST, the uterine myometrium slides were stained with DAPI for 10 min. Finally, an antifade reagent was introduced, and the enrichment of leukocytes in each group was observed using an inverted fluorescence microscope (Nikon, Tokyo, Japan).

### 4.17. Statistical Analyses

The data were analyzed using GraphPad Prism 8.4 software, and the results were presented as the mean ± standard deviation. Significant differences in multiple-comparisons tests were estimated using one-way analysis of variance (ANOVA) with Tukey’s HSD test. Statistical significance was defined as *p* < 0.05.

## 5. Conclusions

Overall, burdock fructooligosaccharide, a natural polysaccharide extracted from traditional Chinese medicine, demonstrates potential in preventing PTL. Its efficacy may be attributed to its ability to act as an antagonist of TLR4, thereby downregulating the expression of its downstream genes, attenuating the release of inflammatory cytokines/chemokines, reducing the infiltration of inflammatory cells into the myometrium, and even mediating the regulatory effects of estrogen and progesterone to effectively protect the animal model from inflammation-induced PTL. Therefore, incorporating BFO as a primary or adjunctive component could offer novel and safer therapeutic insights for developing clinical interventions for inflammation-related PTL, with minimal side effects for mothers and fetuses.

## Figures and Tables

**Table 1:** The protective effects of different concentrations of BFO on LPS-induced preterm birth.

Groups	Preterm Birth %	Average Length from LPS Injection to Birth of the First Pup (h)	Average Length of Pregnancy/Day	Survival Rate of Pups%
PBS + PBS	0	96.2 ± 3.1	19.50 ± 0.2	97.5 ± 5.6
50 μg/kg mouse LPS + PBS	100	20.5 ± 0.9 *	16.35 ± 0.1 *	/
30 μg/g mouse BFO + PBS	0	97.2 ± 2.6 ^##^	19.55 ± 0.1 ^##^	100
50 μg/kg mouse LPS + 15 mg/kg mouse BFO	100	20.25 ± 1.1	16.34 ± 10.1	/
50 μg/kg mouse LPS + 30 mg/kg mouse BFO	42.86	20.03 ± 1.1	16.35 ± 6.5	87.83 ± 11.3 *
		102.2 ± 7.5 ^##^	19.76 ± 0.3 ^##^	
50 μg/kg mouse LPS + 60 mg/kg mouse BFO	28.57	28.2 ± 7.4 ^#^	16.67 ± 6.8	93.30 ± 7.8
		105.5 ± 6.9 ^##^	19.89 ± 0.3 ^##^	
50 μg/kg mouse LPS + 60 mg/kg mouse AMP	71.43	23.62 ± 2.9	16.48 ± 6.2	96.67 ± 9.1
		102.8 ± 6.0 ^##^	19.78 ± 0.2 ^##^	
50 μg/kg mouse LPS + 60 mg/kg mouse CPP	100	21.18 ± 1.2	16.38 ± 0.1	/

Note: The average length from LPS injection to the delivery of the first pup was calculated exclusively for prematurely pregnant mice. The average length of pregnancy refers to the number of the days from the detection of vaginal thrombi to the delivery of the first pup, with the data collected separately for both preterm (upper row) and full-term (lower row) mice in the treatment group. Survival rate of pups is only determined by the number of pups born reaching full term. * *p* ≤ 0.05 vs. control group; ^#^ *p* ≤ 0.05; ^##^ *p* ≤ 0.01 vs. LPS group with one-way ANOVA with Tukey’s HSD test. *N* = 7.

**Table 2:** The protective effects of repeated injection of BFO on LPS- induced preterm birth.

Groups	Preterm Birth %	Average Length from LPS Injection to Birth of the First Pup/h	Average Length of Pregnancy/Day	Survival Rate of Pups %
PBS + PBS + PBS	0	96.2 ± 3.1	19.50 ± 0.2	96.17 ± 6.6
50 μg/kg mouse LPS + PBS + PBS	100	20.24 ± 0.7 **	16.34 ± 0.1 **	/
PBS 30 mg/kg mouse BFO + 30 mg/kg mouse BFO	0	97.92 ± 5.2 ^##^	19.77 ± 0.3 ^##^	95.14 ± 7.6
50 μg/kg mouse LPS + 15 mg/kg mouse BFO + 15 mg/kg mouse BFO	90.91	20.41 ± 0.9	16.35 ± 0.1	/
		96.00		
50 μg/kg mouse LPS + 30 mg/kg mouse BFO + 30 mg/kg mouse BFO	9.09	19.50	19.88 ± 0.3 ^##^	93.98 ± 6.6
		102.5 ± 8.5 ^##^		
50 μg/kg mouse LPS + 60 mg/kg mouse BFO + 60 mg/kg mouse BFO	9.09	20.00	20.2 ± 0.4 ^##^	94.40 ± 6.2
		113.5 ± 7.8 ^##^		

Note: The average length from LPS injection to the delivery of the first pup was calculated exclusively for prematurely pregnant mice. The average length of pregnancy refers to the number of the days from the detection of vaginal thrombi to the delivery of the first pup, with the data collected separately for both preterm (upper line) and full-term (lower line) mice in the treatment group. The survival rate of pups is only determined by the number of pups born reaching full term. ** *p* ≤ 0.01 vs. control group; ^##^ *p* ≤ 0.01 vs. LPS group with one-way ANOVA with Tukey’s HSD test. *N* = 11.

## Data Availability

The complete dataset generated and analyzed during the course of this study has been included in this article. For any further inquiries, please contact the corresponding author.

## Supplementary Materials

The supporting information can be downloaded at: https://www.mdpi.com/article/10.3390/ijms26062659/s1.

## Abbreviations

The following abbreviations are used in this manuscript:

PTL	Preterm labor
BFO	Burdock fructooligosaccharide
CCBs	Calcium channel blockers
DCs	Dendritic Cells
AMP	*Atractylodes Macrocephala* polysaccharide
CPP	*Codonopsis pilosula* polysaccharide
LPS	Lipopolysaccharide
USMC	Uterine smooth muscle cells
PSS	Physiological salt solution
ELISA	Enzyme-Linked Immunosorbent Assay
AUC	Area under the curve
TLR4	Toll-like receptor 4
IF	Immunofluorescence
Icam-1	Intercellular adhesion molecule-1
Vcam-1	Vascular cell adhesion molecule-1
MCP-1	Monocyte chemoattractant protein-1

## References

[1] Sharrow D., Hug L., You D., Alkema L., Black R., Cousens S., Croft T., Gaigbe-Togbe V., Gerland P., Guillot M. (2022). Global, regional, and national trends in under-5 mortality between 1990 and 2019 with scenario-based projections until 2030: A systematic analysis by the UN Inter-agency Group for Child Mortality Estimation. Lancet Glob. Health.

[2] Ward V.C., Lee A.C.C., Hawken S., Otieno N.A., Mujuru H.A., Chimhini G., Wilson K., Darmstadt G.L. (2024). Overview of the Global and US Burden of Preterm Birth. Clin. Perinatol..

[3] Gomez-Lopez N., Galaz J., Miller D., Farias-Jofre M., Liu Z., Arenas-Hernandez M., Garcia-Flores V., Shaffer Z., Greenberg J.M., Theis K.R. (2022). The immunobiology of preterm labor and birth: Intra-amniotic inflammation or breakdown of maternal–fetal homeostasis. Reproduction.

[4] Taylor J., Sharp A., Rannard S.P., Arrowsmith S., McDonald T.O. (2023). Nanomedicine strategies to improve therapeutic agents for the prevention and treatment of preterm birth and future directions. Nanoscale Adv..

[5] Miller F.A., Sacco A., David A.L., Boyle A.K. (2023). Interventions for Infection and Inflammation-Induced Preterm Birth: A Preclinical Systematic Review. Reprod. Sci..

[6] Habelrih T., Augustin T.L., Mauffette-Whyte F., Ferri B., Sawaya K., Côté F., Gallant M., Olson D.M., Chemtob S. (2024). Inflammatory mechanisms of preterm labor and emerging anti-inflammatory interventions. Cytokine Growth Factor Rev..

[7] Keelan J.A. (2018). Intrauterine inflammatory activation, functional progesterone withdrawal, and the timing of term and preterm birth. J. Reprod. Immunol..

[8] Wilson A., Hodgetts-Morton V.A., Marson E.J., Markland A.D., Larkai E., Papadopoulou A., Coomarasamy A., Tobias A., Chou D., Oladapo O.T. (2022). Tocolytics for delaying preterm birth: A network meta-analysis (0924). Cochrane Database Syst. Rev..

[9] Neilson J.P., West H.M., Dowswell T. (2014). Betamimetics for inhibiting preterm labour. Cochrane Database Syst. Rev..

[10] Bhati T., Ray A., Arora R., Siraj F., Parvez S., Rastogi S. (2023). Galectins are critical regulators of cytokine signalling at feto-maternal interface in infection-associated spontaneous preterm birth. Placenta.

[11] Griggs K.M., Hrelic D.A., Williams N., McEwen-Campbell M., Cypher R. (2020). Preterm Labor and Birth: A Clinical Review. MCN Am. J. Matern./Child Nurs..

[12] Bookstaver P.B., Bland C.M., Griffin B., Stover K.R., Eiland L.S., McLaughlin M. (2015). A Review of Antibiotic Use in Pregnancy. Pharmacother. J. Hum. Pharmacol. Drug Ther..

[13] Ichihara Y., Suga K., Fukui M., Yonetani N., Shono M., Nakagawa R., Kagami S. (2020). Serum biotin level during pregnancy is associated with fetal growth and preterm delivery. J. Med. Investig..

[14] Mohammed A.S.A., Naveed M., Jost N. (2021). Polysaccharides; Classification, Chemical Properties, and Future Perspective Applications in Fields of Pharmacology and Biological Medicine (A Review of Current Applications and Upcoming Potentialities). J. Polym. Environ..

[15] Yue Z., Bocheng Y., Zhaoyu W., Mingjing L., Wei Z. (2020). Natural Polysaccharides with Immunomodulatory Activities. Mini-Rev. Med. Chem..

[16] Fang D.-N., Zheng C.-W., Ma Y.-L. (2023). Effectiveness of *Scutellaria baicalensis* Georgi root in pregnancy-related diseases: A review. J. Integr. Med..

[17] Guo Z., Li Q., Wang G., Dong F., Zhou H., Zhang J. (2014). Synthesis, characterization, and antifungal activity of novel inulin derivatives with chlorinated benzene. Carbohydr. Polym..

[18] Versluys M., Porras-Domínguez J.R., Voet A., Struyf T., Van den Ende W. (2024). Insights in inulin binding and inulin oligosaccharide formation by novel multi domain endo-inulinases from Botrytis cinerea. Carbohydr. Polym..

[19] Fernández-Lainez C., Akkerman R., Oerlemans M.M.P., Logtenberg M.J., Schols H.A., Silva-Lagos L.A., López-Velázquez G., de Vos P. (2022). β(2→6)-Type fructans attenuate proinflammatory responses in a structure dependent fashion via Toll-like receptors. Carbohydr. Polym..

[20] Zeng F., Li Y., Zhang X., Shen L., Zhao X., Beta T., Li B., Chen R., Huang W. (2024). Immune regulation and inflammation inhibition of *Arctium lappa* L. polysaccharides by TLR4/NF-κB signaling pathway in cells. Int. J. Biol. Macromol..

[21] Meng Y., Ma Q., Xu X., Feng L., Chen Q., Chen Y., Li Z., Liu C., Chen K. (2023). Burdock fructooligosaccharide ameliorates the hypercholesterolemia and vascular inflammation in mice by regulating cholesterol homeostasis and anti-inflammatory properties. J. Funct. Foods.

[22] Ma Q., Zhang X., Xu X., Lu Y., Chen Q., Chen Y., Liu C., Chen K. (2023). Long-term oral administration of burdock fructooligosaccharide alleviates DSS-induced colitis in mice by mediating anti-inflammatory effects and protection of intestinal barrier function. Immun. Inflamm. Dis..

[23] Li X., Rao Z., Xie Z., Qi H., Zeng N. (2022). Isolation, structure and bioactivity of polysaccharides from *Atractylodes macrocephala*: A review. J. Ethnopharmacol..

[24] Ma K., Yi X., Yang S.-T., Zhu H., Liu T.-Y., Jia S.-S., Fan J.-H., Hu D.-J., Lv G.-P., Huang H. (2024). Isolation, purification, and structural characterization of polysaccharides from *Codonopsis pilosula* and its therapeutic effects on non-alcoholic fatty liver disease in vitro and in vivo. Int. J. Biol. Macromol..

[25] Jiang J., Xu J., Cai L., Man L., Niu L., Hu J., Sun T., Zheng X. (2021). Major depressive symptoms in breast cancer patients with ovarian function suppression: A cross-sectional study comparing ovarian ablation and gonadotropin-releasing hormone agonists. BMC Psychiatry.

[26] Meng X., Kuang H., Wang Q., Zhang H., Wang D., Kang T. (2023). A polysaccharide from Codonopsis pilosula roots attenuates carbon tetrachloride-induced liver fibrosis via modulation of TLR4/NF-κB and TGF-β1/Smad3 signaling pathway. Int. Immunopharmacol..

[27] Wang R., Shan H., Zhang G., Li Q., Wang J., Yan Q., Li E., Diao Y., Wei L. (2022). An inulin-type fructan (AMP1-1) from Atractylodes macrocephala with anti-weightlessness bone loss activity. Carbohydr. Polym..

[28] Meng Y., Xu Y., Chang C., Qiu Z., Hu J., Wu Y., Zhang B., Zheng G. (2020). Extraction, characterization and anti-inflammatory activities of an inulin-type fructan from Codonopsis pilosula. Int. J. Biol. Macromol..

[29] Zierden H.C., Shapiro R.L., DeLong K., Carter D.M., Ensign L.M. (2021). Next generation strategies for preventing preterm birth. Adv. Drug Deliv. Rev..

[30] Swarray-Deen A., Sepenu P., Mensah T.E., Osei-Agyapong J., Sefogah P.E., Appiah-Sakyi K., Ahmed B., Konje J.C. (2024). Preterm birth in low-middle income Countries. Best Pract. Res. Clin. Obstet. Gynaecol..

[31] Yu Y., Shen M., Song Q., Xie J. (2018). Biological activities and pharmaceutical applications of polysaccharide from natural resources: A review. Carbohydr. Polym..

[32] Gupta N., Jangid A.K., Pooja D., Kulhari H. (2019). Inulin: A novel and stretchy polysaccharide tool for biomedical and nutritional applications. Int. J. Biol. Macromol..

[33] Dobrange E., Peshev D., Loedolff B., Van den Ende W. (2019). Fructans as immunomodulatory and antiviral agents: The case of Echinacea. Biomolecules.

[34] Zhang X.-J., Liu S.-F., Lu Y., Wang J.-Y., Chen K.-S. (2019). Immunomodulatory activity of a fructooligosaccharide isolated from burdock roots. RSC Adv..

[35] Siricilla S., Hansen C.J., Rogers J.H., De D., Simpson C.L., Waterson A.G., Crockett S.L., Boatwright N., Reese J. (2023). Arrest of mouse preterm labor until term delivery by combination therapy with atosiban and mundulone, a natural product with tocolytic efficacy. Pharmacol. Res..

[36] Murillo C., Migliorelli F., Nieto C., Martínez H., Rueda C., Bermejo R., Corrales A., Palacio M. (2023). A multi-centre, open-label, prospective, observational study to assess the safety of a nifedipine oral solution in the treatment of preterm labor. Clínica E Investig. En Ginecol. Y Obstet..

[37] Barton G.M., Medzhitov R. (2002). Control of adaptive immune responses by Toll-like receptors. Curr. Opin. Immunol..

[38] Thornton J.M., Ramphul M. (2023). Mechanisms and management of normal labour. Obstet. Gynaecol. Reprod. Med..

[39] Green E.S., Arck P.C. (2020). Pathogenesis of preterm birth: Bidirectional inflammation in mother and fetus. Semin. Immunopathol..

[40] Basith S., Manavalan B., Lee G., Kim S.G., Choi S. (2011). Toll-like receptor modulators: A patent review (2006-2010). Expert Opin. Ther. Pat..

[41] Farias-Jofre M., Romero R., Galaz J., Xu Y., Miller D., Garcia-Flores V., Arenas-Hernandez M., Winters A.D., Berkowitz B.A., Podolsky R.H. (2023). Blockade of IL-6R prevents preterm birth and adverse neonatal outcomes. eBioMedicine.

[42] Saito S., Kasahara T., Kato Y., Ishihara Y., Ichijo M. (1993). Elevation of amniotic fluid interleukin 6 (IL-6), IL-8 and granulocyte colony stimulating factor (G-CSF) in term and preterm parturition. Cytokine.

[43] McGoldrick E., Stewart F., Parker R., Dalziel S.R. (2020). Antenatal corticosteroids for accelerating fetal lung maturation for women at risk of preterm birth. Cochrane Database Syst. Rev..

[44] Crowther C.A., Middleton P.F., Voysey M., Askie L., Pryde P.G., Marret S., Doyle L.W. (2017). Assessing the neuroprotective benefits for babies of antenatal magnesium sulphate: An individual participant data meta-analysis. PLOS Med..

[45] Condon J.C., Kyathanahalli C., Anamthathmakula P., Jeyasuria P. (2020). Estrogen/estrogen receptor action and the pregnant myometrium. Curr. Opin. Physiol..

[46] Brown A.G., Leite R.S., Strauss Iii J.F. (2004). Mechanisms Underlying “Functional” Progesterone Withdrawal at Parturition. Ann. New York Acad. Sci..

[47] Nakaya M., Tachibana H., Yamada K. (2006). Effect of Estrogens on the Interferon-γ Producing Cell Population of Mouse Splenocytes. Biosci. Biotechnol. Biochem..

[48] Huang Q., Jin X., Li P., Zheng Z., Jiang Y., Liu H. (2021). Elevated inflammatory mediators from the maternal-fetal interface to fetal circulation during labor. Cytokine.

[49] Huang Q., Ye A., Li P., Bao J., Garfield R.E., Liu H. (2022). Nicotine ameliorates inflammatory mediators in RU486 induced preterm labor model through activating cholinergic anti-inflammatory pathway. Cytokine.

[50] Tong M., Abrahams V.M. (2020). Neutrophils in preterm birth: Friend or foe?. Placenta.

[51] Chao H.-H., Li L., Gao X., Wang C., Yue W. (2019). CXCL12 expression in aborted mouse uteri induced by IFN-γ: Potential anti-inflammatory effect involves in endometrial restoration after abortion in mice. Gene.

[52] Garcia-Flores V., Romero R., Peyvandipour A., Galaz J., Pusod E., Panaitescu B., Miller D., Xu Y., Tao L., Liu Z. (2023). A single-cell atlas of murine reproductive tissues during preterm labor. Cell Rep..

[53] Gao A., Li L., Yan F., Lei Y., Chen J., Wu L., Ye J. (2021). Nile tilapia CXCR4, the receptor of chemokine CXCL12, is involved in host defense against bacterial infection and chemotactic activity. Dev. Comp. Immunol..

[54] Wang Y., Zhang N., Kan J., Zhang X., Wu X., Sun R., Liu J., Qian C., Jin C. (2019). Structural characterization of water-soluble polysaccharide from Arctium lappa and its effects on colitis mice. Carbohydr. Polym..

[55] Salminen A., Vuolteenaho R., Paananen R., Ojaniemi M., Hallman M. (2012). Surfactant protein D modulates levels of IL-10 and TNF-α in intrauterine compartments during lipopolysaccharide-induced preterm birth. Cytokine.

[56] Li K., Li S., Du Y., Qin X. (2020). Screening and structure study of active components of Astragalus polysaccharide for injection based on different molecular weights. J. Chromatogr. B.

[57] Xu X., Shao T., Meng Y., Liu C., Zhang P., Chen K. (2023). Immunomodulatory mechanisms of an acidic polysaccharide from the fermented burdock residue by *Rhizopus nigricans* in RAW264.7 cells and cyclophosphamide-induced immunosuppressive mice. Int. J. Biol. Macromol..

[58] Zhang Y., Tang W., Zheng Z., Nie G., Zhan Y., Mu X., Liu Y., Wang K. (2023). Metabolic degradation of polysaccharides from Lentinus edodes by Kupffer cells via the Dectin-1/Syk signaling pathway. Carbohydr. Polym..

