# Supplementary material for: The Protective Effects of Burdock Fructooligosaccharide on Preterm Labor Through Its Anti-Inflammatory Action
Source: Int J Mol Sci. 2025 Mar 15;26(6):2659. doi: 10.3390/ijms26062659 (PMC11942195; doi:10.3390/ijms26062659)
Supplement: Supplementary file 1 [file ijms-26-02659-s001.zip › ijms-3458003-supplementary.pdf]

SUPPLEMENTARY INFORMATION

The Protective Effects of Burdock Fructooligosaccharide on Preterm Labor Through its Anti-Inflammatory Action

Qunfei Ma [1,†], Ruoheng Du [1,†], Peihua Long [1], Kaiyi Sun [1], Youxia Wang [1], Ye Yang [1], Xinyu Shen [1] and Lu Gao [1,2,*]

[1] Department of Physiology, Naval Medical University, Shanghai, 200433, PR China
[2] Shanghai Key Laboratory for Assisted Reproduction and Reproductive Genetics, Shanghai, 200120, PR China
* Lu Gao: lu.gao@smmu.edu.cn; Tel.: +86-21-81870980
† These authors contributed equally to this work.

Supplementary Table S1. A sequence of genetic primers applicable to the mice uterine muscle cells

Gene name	Forward primer (5′-3′)	Reverse primer (3′-5′)
IL-1β	TGGACCTTCCAGGATGAGGACA	GTTCATCTCGGAGCCTGTAGTG
IL-6	TACCACTTCACAAGTCGGAGGC	CTGCAAGTGCATCATCGTTGTTC
TNF-α	ATGGCCCAGACCCTCACACTCA	TGGTGGTTTGCTACGACGTGGG
Ccl2	GCTACAAGAGGATCACCAGCAG	GTCTGGACCCATTCCTTCTTGG
Ccl5	CCTGCTGCTTTGCCTACCTCTC	ACACACTTGGCGGTTCCTTCGA
Cxcl2	CATCCAGAGCTTGAGTGTGACG	GGCTTCAGGGTCAAGGCAAACT
Cxcl12	GGAGGATAGATGTGCTCTGGAAC	AGTGAGGATGGAGACCGTGGTG
IL-10	CGGGAAGACAATAACTGCACCC	CGGTTAGCAGTATGTTGTCCAGC
TGF-β2	TTGTTGCCCTCCTACAGACTGG	GTAAAGAGGGCGAAGGCAGCAA
MyD88	ACCTGTGTCTGGTCCATTGCCA	GCTGAGTGCAAACTTGGTCTGG
Traf6	TTTCCCTGACGGTAAAGTGCCC	ACCTGGCACTTCTGGAAAGGAC
NF-κB	GCTGCCAAAGAAGGACACGACA	GGCAGGCTATTGCTCATCACAG
Gapdh	CATCACTGCCACCCAGAAGACTG	ATGCCAGTGAGCTTCCCGTTCAG

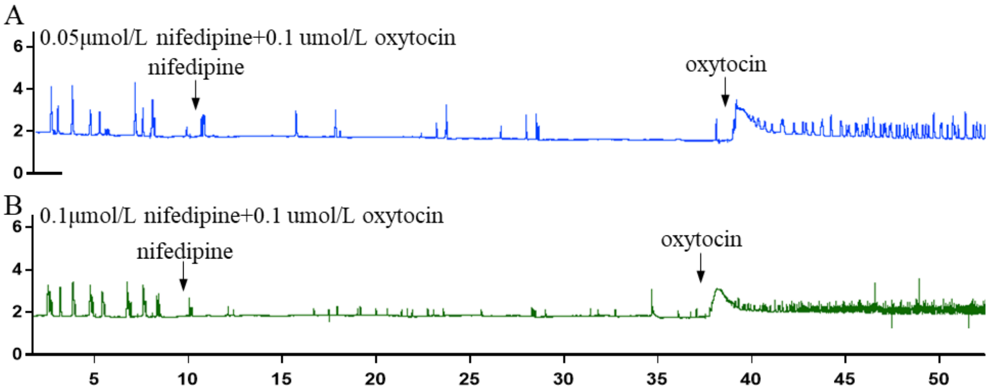

Supplementary Figure S1. Effects of nifedipine on oxytocin-induced myometrium contraction of pregnant mice *in vitro*. The uterine myometrial strips were preconditioned with (A) 0.05µmol/L nifedipine and (B) 0.1µmol/L nifedipine, followed by 0.1µmol/L oxytocin treatment.

We first prepared purified BFO-Tyr through amination reaction, which has a characteristic absorption peak of Tyr at 280nm (Figure S3A). Then, BFO-Tyr was subjected to an affinity reaction with FITC and separated and purified by Sephadex G-50. Two fluorescence peaks were found, namely peak1 and peak2 (Figure S3B). Wherein peak1 had characteristic absorption peaks of Tyr and FITC near 280 nm and 480 nm, respectively (Figure S3C), while Peak2 did not show any obvious characteristic absorption peaks (Figure S3D), indicating that Peak1 was BFO-Tyr-FITC. The FT-IR spectrum of BFO-Tyr-FITC showed a similar curve to that of BFO, indicating that the labeled component has no distinct effect on the skeleton structure of the polysaccharide (Figure S3E).

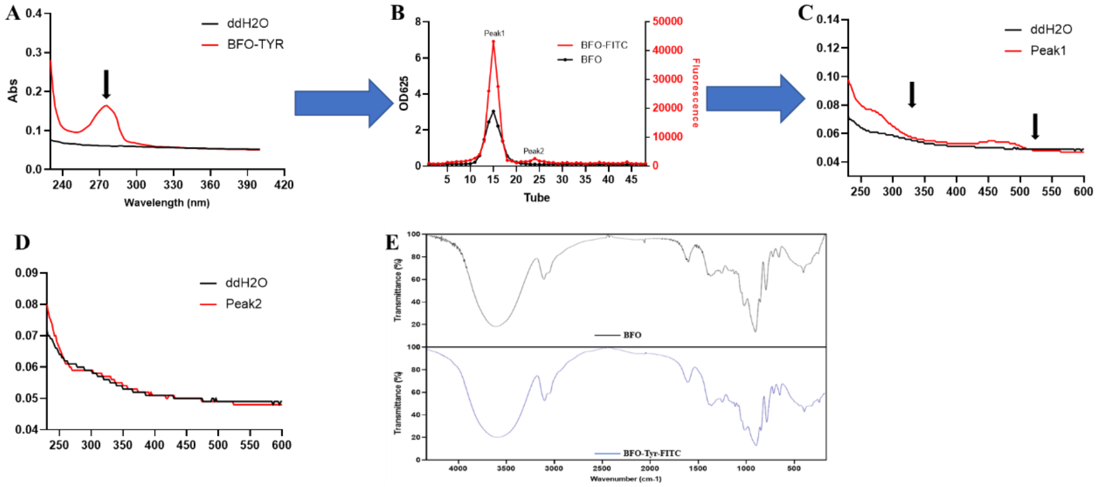

Supplementary Figure S2. The fluorescence labeling process of BFO. (A) A spectral scan of BFO-Tyr at wavelength 230-400 nm was conducted. (B) The G-50 gel chromatographic elution peak of BFO-Tyr-FITC was observed. The red peak indicated the fluorescence absorption, while the black peak indicated the polysaccharide detection. A 230-600nm spectral scan of the (C) Peak1 and (D) Peak2 were performed. And (E) the FT-IR spectrum of BFO (up) and BFO-Tyr-FITC (down) were obtained.
